# An Automated Curriculum to Support Behavioral Health Counseling Among Pediatric Residents: Usability Study

**DOI:** 10.2196/77438

**Published:** 2026-04-22

**Authors:** Liam Fleck, Rachel Herbst, Andrea Meisman, Thomas Talbot, Michael Glisson, Max Remington, Micah Goldson, Francis Real

**Affiliations:** 1 Cincinnati Children's Hospital Medical Center, Cincinnati, OH, United States; 2 University of Cincinnati, 3333 Burnet Ave, Cincinnati, OH, 45229, United States, 1 4847163867; 3 USC Institute for Creative Technologies, Los Angeles, CA, United States; 4 BreakAway, Ltd, Hunt Valley, MD, United States

**Keywords:** virtual reality, artificial intelligence, AI, medical education, health communication, behavioral health, motivational interviewing

## Abstract

**Background:**

Behavioral health concerns are common in pediatric practice, with pediatricians reporting a lack of skills related to providing effective behavioral management strategies to parents. A prior human-facilitated, screen-based virtual reality training curriculum proved effective in enhancing behavioral health communication skills among pediatric residents. However, barriers to spread and scale of the curriculum included the need for human facilitation.

**Objective:**

This study explored the usability of an automated virtual reality–based behavioral health anticipatory guidance curriculum for pediatric residents.

**Methods:**

Through a partnership with BreakAway Ltd, an automated prototype was developed that required verbalization by users to support progress through simulated scenarios and included the receipt of personalized feedback to enable deliberate practice of communication skills. Usability of the prototype was assessed using mixed methods that included in-person completion of the prototype, a semistructured interview, and completion of survey instruments including the System Usability Scale and the Measurement, Effects, Conditions: Spatial Presence Questionnaire.

**Results:**

Nine individuals completed usability testing. Qualitatively, users indicated that the system was easy to use, realistic, gamified, and likely most helpful for novice learners. Quantitatively, the ease of system usability was rated highly with some limitations related to spatial presence noted.

**Conclusions:**

Usability testing of an automated curriculum to support behavioral health counseling skills in pediatric residents was completed, providing data to support adaptations in preparation for implementation.

## Introduction

### Background

Although pediatricians frequently discuss behavioral health concerns with families [1], they report a lack of training on how to effectively address these concerns using evidence-based approaches and collaborative communication [2,3]. This represents a critical training gap, as the implementation of effective behavioral management strategies by parents to address typical childhood behaviors (eg, tantrums) can mitigate the development of future behavioral health disorders [4]. With approximately 20% of children currently having a diagnosis of a behavioral health disorder [5], identification of training strategies to support future pediatricians’ skills in delivering behavioral health anticipatory guidance (BHAG) is needed [6]. Recent curricula aiming to support BHAG skills in pediatric residents have primarily relied upon experts to deliver content via didactic or case-based discussions, limiting the opportunity for spread and scale of effective interventions [7,8]. Simulation-based medical education in the form of patient actors has been incorporated into training pediatric residents on BHAG; however, limitations related to accessibility, realism, and psychological safety have been reported [9]. Advances in technology offer an opportunity for broad dissemination of theory-based education. Screen-based virtual reality (VR) is one technology that can be leveraged to support BHAG skill development in pediatric residents to optimize pediatric health outcomes.

### Prior Work

We previously developed a novel screen-based VR curriculum to enhance BHAG competencies among pediatric residents through deliberate practice of skills [10-12]. A human facilitator drove both the VR responses of graphical characters in scenarios and provided personalized performance feedback following each simulated scenario. The VR curriculum proved highly acceptable and efficacious in enhancing BHAG and health care communication skills [10,12-14]. During usability testing of the human-facilitated curriculum, residents reported the curriculum as realistic, engaging, practical, appropriately scaffolded, and psychologically safe. They indicated the curriculum was easy to use and reported high levels of immersion and spatial presence within the virtual environment [10]. The spread and scale of this education was limited due to the need for highly trained human facilitators. Artificial intelligence (AI) offers an opportunity to explore how automation may support maintenance and scale of such VR-based educational interventions.

AI allows users to interact with computer systems that use data sources to interpret user inputs and deliver curated responses [15]. The use of AI in medical education and simulation has been limited; prior research indicates limitations related to realism and accuracy [16,17]. Thus, we sought to assess the feasibility and acceptability of an automated curriculum to support BHAG and communication skills in pediatric residents.

## Methods

### Ethical Considerations

This study was reviewed and determined to be exempt by the Cincinnati Children's Hospital Medical Center Institutional Review Board (2023-0314). A waiver of documentation of informed consent was granted; therefore, prior to data collection, participants reviewed a study information sheet, and participation in study procedures indicated their consent. All study team members adhered to institutional procedures governing data access and privacy. Compensation was provided to all participants.

### Curriculum Development

To develop the automated intervention, we partnered with BreakAway Ltd, a game developer that created a conversational interaction system titled Standard Patient Studio (SPS) [18]. SPS uses a cloud-based AI platform in which progression through a simulated scenario is driven by a user’s verbalizations. A multidisciplinary team that included 2 pediatricians, 1 pediatric psychologist, 3 software developers with expertise in AI, and 2 research assistants adapted the human-facilitated VR curriculum to the SPS platform. Procedures included scenario storyboarding with branching logic using the prior human-facilitated VR scenarios as a guide, with iterative scenario development testing by study team members. The final curricular prototype yielded 2 scenarios that replicated the human-facilitated VR curriculum’s learning objectives and used identical verbal statements by the graphical parent. Potential answer options for the learner were displayed on the screen during each scenario to support scaffolded learning and to inform a learner’s spontaneous verbalizations, thereby ensuring progression through a scenario. The answer option most closely aligned with the user’s verbalizations was selected by the natural language understanding system. This system categorizes user input via a medical taxonomy reference that assigns an appropriate patient action. In prior trials, this system has demonstrated a 92% appropriate response rate [19]. On the basis of the selected answer option, the user received immediate feedback (ie, excellent, acceptable, or poor response) via color-coded emojis that were previously embedded in the SPS platform. Upon scenario completion, users received feedback indicating why an answer selection was preferred or not preferred. The feedback mirrored the human-facilitated feedback by identifying the specific BHAG and motivational interviewing skills demonstrated during a scenario and where there were missed opportunities to demonstrate such skills. A low performance score required users to repeat the scenario, which mirrored the deliberate practice approach used for the human-facilitated VR training.

### Usability Testing

Usability testing, a critical component of intervention development, which seeks to systematically evaluate the extent to which an intervention achieves its intended purpose by engaging experts to identify weaknesses, occurred using mixed methods [20,21]. Health care team members, including senior faculty, psychologists, simulation educators, and pediatric residents, were purposefully sampled to pilot the curriculum. Usability testing occurred in person, where users were asked to “think aloud,” verbalizing their perspectives as they navigated the automated curriculum [22]. Following completion, participants underwent a semistructured interview to explore their overall impressions (Textbox 1). Interviews were recorded, transcribed, and analyzed by 2 raters (AM and FR) using the rigorous and accelerated data reduction (RADAR) technique, a rapid qualitative analysis approach [23]. RADAR uses a 5-step approach to data reduction through the iterative refinement of data tables and is well suited for studies with narrow research questions. The raters ensured consensus on reduction decisions at each step. Trustworthiness of the qualitative data was also supported through the use of an interview guide that generated descriptive responses (credibility), multiple analyzers and peer debriefing (dependability), and recording of detailed procedures (confirmability) [24]. Participants also completed the System Usability Scale (SUS) [25] to examine the platform’s ease of use and a subset of items from the Measurement, Effects, Conditions: Spatial Presence Questionnaire (MEC-SPQ) [26] to assess for immersion in the virtual environment.

Textbox 1. **Interview guide.**

Usability interview guide items

- What are your overall impressions of the automated curriculum?
- How easy or difficult was the curriculum to use? Tell me more.
- How was your interaction with the avatar characters?
  - What would you change?

- What were your impressions of the feedback you received after the simulation?
  - Did it seem to accurately reflect your performance?
  - How easy or difficult was it to interpret?

- What one change would you make to the automated curriculum?
- Would you recommend the automated curriculum for learners? Why or why not?

## Results

A total of 9 individuals, including 2 pediatric psychologists, 3 senior pediatric faculty, 2 pediatric residents, and 2 simulation educators, underwent testing during a 1-week period in August 2024. Participants had a mean age of 36 (SD 7.9) years and were mostly women (n=8, 89%), White (n=8, 89%), and non-Hispanic (n=9, 100%).

### Qualitative

Of the 9 participants, 8 (89%) provided qualitative data. Participants described the scenarios as relevant and realistic. Overall, they reported that the system was easy to use and correctly coded their verbalizations. The study team observed, and participants reported, that there was a learning curve associated with using the online microphone. Participants indicated appreciation for the nuanced answer options and level of difficulty, although some participants desired decreased scaffolding and increased free agency. Some participants described a game-based experience, attributed to the immediate feedback in the form of color-differentiated emojis. Some viewed the game mechanics integration positively, while others felt it detracted from realism. All participants (n=8) indicated that they would recommend the curriculum for learners. Due to the inclusion of game-based elements and the select option format, some participants believed the curriculum was best suited for novice learners (eg, medical students and residents) rather than practicing pediatricians. All participants indicated that the feedback accurately reflected their performance. When asked what one change participants would make to the automated curriculum, 3 (38%) requested more free agency and less scaffolding during the simulations, 2 (25%) wanted shorter answer options, 2 (25%) wanted more variability in terms of graphical character responses, and 1 (13%) wanted more specific feedback regarding incorrect answer selections.

Table 1 includes exemplar quotes informing these data interpretations.

**Table 1.** Principal themes and supporting quotes.

Theme	Supporting quotes
Easy to use	“I thought it was very easy. I had no problems using the system and getting through the scenario.” [ID 26] “It’s very self-explanatory, and you just have to like push a button. It’s not super high, not high-tech in a way that I’m like I have no clue what to do with this.” [ID 21]
Realistic scenarios	“I think it’s a practical way, some of the things that the parents said are very realistic and real life that we hear all the time, so I thought that was realistic.” [ID 24] “I felt the situation was very realistic, and it felt like the, it just, it felt accurate. That’s my biggest reaction. These things come up all the time, and kids do have tantrums in the office all the time, so this is very relevant.” [ID 26]
Accurate character responses	“I feel like she responded appropriately to what I said.” [ID 22] “I think I would say it feels representative in some ways of what like parents might say.” [ID 27]
Game-based design	“I felt more like it was like a game to find the best answer than an actual interaction with the person. It felt more like a trivia game.” [ID 26] “It [the feedback] felt like a score, like a game and a score.” [ID 27]
Potential for more realistic characters	“I would like the room to look more realistic, and I’d like Phyllis [the graphical parent] to look more realistic, and I’d like Alex [the graphical child] to actually be with Phyllis and not a pop-out video.” [ID 30] “I mean, if it looked more realistic, obviously, that would be nice. And her voice sounds a little bit monotone. Like if it could sound a little bit more natural, then I think I would feel more bought in that it feels like a more realistic patient interaction.” [ID 22]
Accurate and clear feedback	“I like this [feedback] page, especially because it breaks it up, and it gives specific feedback. I guess sometimes I didn’t pick the most egregious answers, so I didn’t always get like the bad feedback. But I do like that you could see kind of what the other choices are, and you could kind of see what the scale is.” [ID 29] “I like that it’s relatively clear [the feedback]. Obviously, you know, there’s the green, yellow, red. Green is good. Yellow is kind of that middle of the road. Red is bad.” [ID 23]
Most helpful for novice learners	“I can see how it could have a lot of value for resident training and definitely helping residents get more experience.” [ID 27] “I think I would, especially for like interns or early learners because, I mean, it’s low stakes with the avatar. And I also like the microphone aspect of it, because I think speaking the words instead of just where you’ve been clicking goes a long way.” [ID 29]

### Quantitative

The overall SUS mean score among participants was 78 (SD 9.8; range 65.0-92.5), indicating good to excellent usability of the system [27]. On the MEC-SPQ, 44% (4/9) of participants agreed or strongly agreed that it felt like they actually participated in the action of the simulation. Only 11% (1/9) agreed or strongly agreed that they felt as though they were physically present in the environment. (Table 2)

**Table 2.** Quantitative outcome metrics assessing system usability (System Usability Scale [25]) and spatial presence (adapted Measurement, Effects, Conditions: Spatial Presence Questionnaire [MEC-SPQ] [26]), which both use a 5-point Likert scale from strongly disagree (score=1) to strongly agree (score=5).

Items	Score, mean (SD)
System Usability Scale	
I think that I would like to use this system frequently	3.6 (0.9)
I found the system unnecessarily complex	1.9 (1.1)
I thought the system was easy to use	4.1 (0.6)
I think that I would need the support of a technical person to be able to use this system	1.4 (0.5)
I found the various functions in this system were well integrated	3.8 (0.8)
I thought there was too much inconsistency in this system	1.9 (0.9)
I would imagine that most people would learn to use this system very quickly	4.3 (0.5)
I found the system very cumbersome to use	1.6 (0.5)
I felt very confident using the system	4.0 (0.7)
I needed to learn a lot of things before I could get going with this system	1.7 (0.5)
Total	78.3 (9.8)
MEC-SPQ (subset)	
The virtual reality experience captured my senses (ie, it held my attention)	3.3 (1.0)
I dedicated myself completely to the virtual reality experience. (ie, I was not distracted)	3.4 (1.0)
I was able to make a good estimate of the size of the presented space	3.2 (0.8)
I felt as though I was physically present in the environment of the simulation	2.6 (1.0)
It seemed as though I actually took part in the action of the simulation	3.1 (0.9)
The objects in the simulation gave me the feeling that I could do things with them	2.4 (1.0)
Even now, I still have a concrete mental image of the spatial environment	3.6 (1.0)

## Discussion

### Principal Results

Adaptation of a human-facilitated VR curriculum on BHAG was successfully automated through the use of the SPS platform developed by BreakAway Ltd. Qualitative and quantitative data indicated that users could easily navigate the platform and interact with its components. Compared with the human-facilitated VR curriculum [10], users reported less spatial presence in the virtual environment. However, users still described the automated training as realistic and relevant. Users also endorsed a game-based aspect to the training experience, which was not reported with the prior human-facilitated VR curriculum [10]. Overall, users indicated that the current automated training might be most appropriate for novice learners. Our decision to scaffold the learning experience by displaying answer options supported reliable progression through the scenarios and safeguarded against misclassification of verbalizations, which can occur when using generative AI models [17,28-30]. However, more advanced learners may benefit from removing scaffolded answer options at a limited number of interaction points.

### Limitations

This study had several limitations. First, our small, purposeful sample, ideal for usability testing [31-33] and intervention development, limited generalizability. However, recent literature by Guest et al [31,32] indicates that 6 to 7 interviews are likely sufficient to support theme generation for homogenous samples such as ours. Second, the rapid qualitative analysis method may have limited exploration of interview data. However, the RADAR technique is well suited for research questions that are narrow in scope [23].

### Comparison with Prior Work

Behavioral health medical education lacks effective, innovative training curricula necessary to develop practice competencies [3]. By removing the need for a human facilitator or patient actors, AI-based communication training provides an opportunity for spread and scale [16,34]. Additional research is needed on how to best incorporate AI into education interventions to optimize learning outcomes that support behavior change. We specifically sought to align the automated curriculum with deliberate practice principles, as this served as the theoretical foundation for the prior human-facilitated VR curriculum [11]. Deliberate practice is a personal, goal-oriented approach to training that focuses on attempting a behavior (eg, providing BHAG), receiving immediate feedback, and then repeating it until demonstrating skill mastery. Consistent with this theoretical framework, the automated curriculum provided a platform for rehearsal of specific verbiage, feedback based on each interaction point, repetition of skills over the two scenarios, and the opportunity to repeat scenarios. Aligning AI-based educational interventions with evidence-based adult learning theories will allow us to establish a literature base on how AI might most effectively support learning. This is particularly important for the topic of behavioral health, as recent studies describing curricular interventions often do not specifically indicate a theoretical foundation for the work [8,35,36]. As AI becomes integrated routinely into training, we should consider how we might best facilitate trust for learners interacting with AI systems as well as provide transparency regarding AI algorithms and source data [37].

### Conclusions

Our study demonstrated the feasibility of developing an automated curriculum to support BHAG skills in pediatric residents through collaboration with an experienced industry partner. Automation of training curricula adds value by enhancing the capacity for scalability by reducing dependence on human facilitation. As such, automation also decreases the costs for participation, supporting equity among learners in accessing novel, evidence-based education. Next steps include updating the curriculum based on usability testing and implementing it among pediatric residents to assess its acceptability and effectiveness among that population and to explore how its outcomes compare to other training modalities, such as human-facilitated VR interventions. An evaluation of implementation strategies will also be critical to understand how such novel education can be integrated into resident curricula. Moreover, given the varying perceptions related to the game-based elements incorporated into the intervention, further exploration of how such game-based features impact attitudes and learning outcomes is warranted. We also plan to add an orientation regarding learning goals prior to intervention completion to align with best practices for promoting a constructive digital feedback environment [38]. In the future, we aim to refine the automated curriculum, including adjusting the level of free agency and gamified elements, to support learning across the continuum of health care providers.

## Abbreviations

AI: artificial intelligence
BHAG: behavioral health anticipatory guidance
MEC-SPQ: Measurement, Effects, Conditions: Spatial Presence Questionnaire
RADAR: rigorous and accelerated data reduction
SPS: Standard Patient Studio
SUS: System Usability Scale
VR: virtual reality
